# Highly Efficient Energy Transfer from Silicon to Erbium in Erbium-Hyperdoped Silicon Quantum Dots

**DOI:** 10.3390/nano13020277

**Published:** 2023-01-09

**Authors:** Kun Wang, Qiang He, Deren Yang, Xiaodong Pi

**Affiliations:** 1 State Key Laboratory of Silicon and Advanced Semiconductor Materials & School of Materials Science and Engineering, Zhejiang University, Hangzhou 310027, China; 2 Institute of Advanced Semiconductors & Zhejiang Provincial Key Laboratory of Power Semiconductor Materials and Devices, Hangzhou Innovation Center, Zhejiang University, Hangzhou 311215, China

**Keywords:** nonthermal plasma, Er-hyperdoped Si QDs, efficiency of energy transfer, coupling constant, strong coupling

## Abstract

Erbium-doped silicon (Er-doped Si) materials hold great potential for advancing Si photonic devices. For Er-doped Si, the efficiency of energy transfer (*η_ET_*) from Si to Er^3+^ is crucial. In order to achieve high *η_ET_*, we used nonthermal plasma to synthesize Si quantum dots (QDs) hyperdoped with Er at the concentration of ~1% (i.e., ~5 × 10^20^ cm^−3^). The QD surface was subsequently modified by hydrosilylation using 1-dodecene. The Er-hyperdoped Si QDs emitted near-infrared (NIR) light at wavelengths of ~830 and ~1540 nm. An ultrahigh *η_ET_* (~93%) was obtained owing to the effective energy transfer from Si QDs to Er^3+^, which led to the weakening of the NIR emission at ~830 nm and the enhancement of the NIR emission at ~1540 nm. The coupling constant (*γ*) between Si QDs and Er^3+^ was comparable to or greater than 1.8 × 10^−12^ cm^3^·s^−1^. The temperature-dependent photoluminescence and excitation rate of Er-hyperdoped Si QDs indicate that strong coupling between Si QDs and Er^3+^ allows Er^3+^ to be efficiently excited.

## 1. Introduction

Silicon (Si) photonics has been intensively investigated due to its excellent compatibility with Si-based complementary metal oxide semiconductor (CMOS) technologies [1,2]. While monolithic Si-compatible solutions for various devices such as detectors, waveguides, and modulators have been available for years, the development of Si photonics has been impeded by the lack of monolithic energy-efficient and cost-effective light sources [3,4]. Owing to a spectroscopically sharp and environment-stable radiative transition at the wavelength (λ) of 1.54 μm that is highly desired for Si photonics [5], Er-doped Si is an ideal material for the fabrication of light sources in Si photonics. However, the significant thermal quenching of Er-doped bulk Si restricts or even forbids its use in optoelectronic devices [6,7]. Because Er-doped Si QDs can effectively emit at a wavelength of 1.54 μm at room temperature with mild thermal quenching, they have attracted enormous attention [8]. The photoluminescence (PL) of 1.54 μm originates from the transfer from the exciton energy of Si QDs to Er^3+^ and the following inter-4*f* transition of Er^3+^. Hence, the *η_ET_* plays a significant role in the practical applications of Er-doped Si QDs. The *η_ET_* can be described by [9]

(1) $$\eta_{ET}=1-\frac{\tau_{\mathrm{Er}-\mathrm{Si}\;\mathrm{QDs}}}{\tau_{\mathrm{Si}\;\mathrm{QDs}}}$$

where *τ*_Er−Si QDs_ and *τ*_Si QDs_ are the PL lifetime of Er-doped and undoped Si QDs, respectively. H. Rinnert et al. reported Er-doped Si QDs embedded in SiO/SiO_2_ multilayers, whose *η_ET_* was estimated as ~50% [10]. Timoshenko et al. also reported similar *η_ET_* (~50%) for Er-doped Si QDs embedded in SiO/SiO_2_ multilayers [11]. Despite this, earlier studies demonstrated that Er^3+^ was only found in close proximity to Si QDs inside a dielectric matrix, resulting in a system with a low degree of coupling [12]. The weakly coupled system hindered exciton-to-Er^3+^ energy transfer in Er-doped Si QDs because of the variable distance between Si QDs and Er^3+^. Notably, the exciton-to-Er^3+^ energy transfer should be most effective if Er^3+^ is present in the Si QD in order to facilitate the overlap of electron wavefunctions [13]. This motivates the development of Si QDs into which Er^3+^ is incorporated as a dopant.

Here, nonthermal plasma was used to fabricate Er-hyperdoped Si QDs at an atomic concentration of 1% (5 × 10^20^ cm^−3^), surpassing its solubility. Er-hyperdoped Si QDs were found to emit NIR light at 830 and 1540 nm, with the majority of the Er^3+^ located in the subsurface area. As a result of the effective transfer of energy from Si QDs to Er^3+^, the Er-hyperdoped Si QDs exhibited an ultrahigh *η_ET_* of ~93%, emitting at 1540 nm. Meanwhile, the efficient energy transfer from Si QDs to Er^3+^ greatly reduced the 830 nm NIR emission. Additionally, a high effective excitation cross section of Er^3+^ (*σ_Er_*) was obtained, with a value of 1.5 × 10^−17^ cm^2^. Furthermore, the coupling constant (*γ*) between Si QD and Er^3+^ was greater than or equal to 1.8 × 10^−12^ cm^3^·s^−1^. The temperature-dependent photoluminescence and excitation rate of Er-hyperdoped Si QDs indicate that strong coupling between Si QDs and Er^3+^ allows Er^3+^ to be efficiently excited.

## 2. Materials and Methods

### 2.1. Materials

Er(tmhd)_3_ (tris(2,2,6,6-tetramethyl-3,5-heptanedionate)) (99.999%) was purchased from Nanjing Aimouyuan Scientific equipment Co., Ltd. (Nanjing, China). SiH_4_/Ar (20%/80% in volume) was obtained from Linde Electronic & Specialty Gases Co., Ltd. (Suzhou, China). Mesitylene (97%) and 1-dodecene (97%) were purchased from Aladdin (Shanghai, China). Methanol (≥98.5%), hydrofluoric (HF) acid (≥40%), and toluene (≥99.5%) were obtained from J&K Scientific (Beijing, China). Reference solutions of Er (100 µg·mL^−1^ in nitric acid) and Si (1000 µg·mL^−1^ in nitric acid) were purchased from Qingdao Qingyao Biological Engineering Co., Ltd. (Qingdao, China) and Sigma-Aldrich Trading Co., Ltd. (Shanghai, China), respectively.

### 2.2. Synthesis of Er-Hyperdoped Si QDs

**Figure 1:**
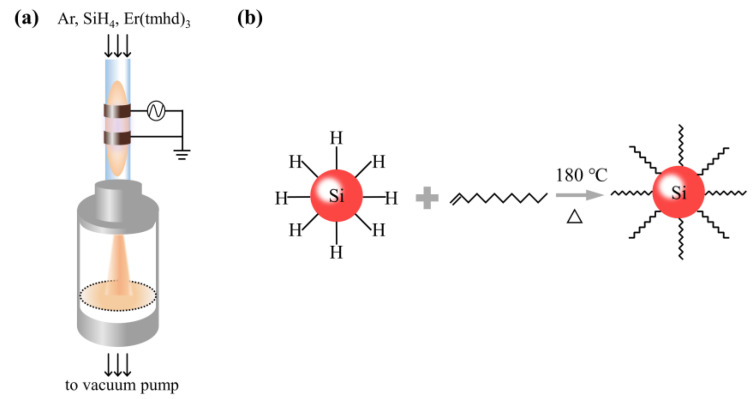
(**a**) Diagram depicting the nonthermal plasma. (**b**) Schematic of the surface hydrosilylation process.

A mechanical pump was used to pump the pressure within the plasma chamber to 8 × 10^−2^ mBar, and a heating strip was used to increase the temperature of the pipeline and the erbium precursor Er(tmhd)_3_ to 200 °C. The plasma chamber was filled with 4.8 sccm of a 20% SiH_4_/Ar mixture and 500 sccm of Ar-loaded Er(tmhd)_3_ (Figure 1a). The pressure of the plasma chamber was adjusted to ~3 mBar. Making use of a matching network and a power source operating at 13.56 MHz, the plasma was generated. The actual output power was stabilized at 70 W. Methanol was used to disperse the powder of Er-hyperhoped Si QDs for surface hydrosilylation (Figure 1b). After that, HF acid was used to strip the surface oxide off. An amount of 15 mL of mesilytene and 5 mL of 1-dodecene were combined, and then the precipitate was added into the mixture after centrifugation. The solution was heated at 180 °C for 3 h in an Ar environment. In order to remove the hydrosilylated Er-hyperdoped Si QDs from the solution, rotary evaporation was used. Finally, the toluene was used to disperse the prepared hydrosilylated Er-hyperdoped Si QDs.

### 2.3. Characterization

An FEI Tecnai G2 F20 S-TWIN (FEI, Hillsboro, OR, USA) was used to capture the TEM images, operating at an acceleration voltage of 200 kV. High-angle annular darkfield scanning transmission electron microscopy (HAADF-STEM) was performed and element map images were obtained using the FEI Titan G2 80-200 (FEI, Hillsboro, OR, USA) operated at an acceleration voltage of 200 kV. X-ray photoelectron spectroscopy (XPS) was used to examine the oxidation states in each sample (Kratos Shimadzu, AXIS Supra, Manchester, UK). Er concentration was calculated using ICP-MS (iCAP6300, Thermo, Waltham, MA, USA). The Shimadzu-7000 (Shimadzu, Kyoto, Japan) was used for the X-ray diffraction (XRD) tests. Raman spectra were obtained to analyze the strain (Alpha300R, WITec, Ulm, Germany). Using FLS1000 PL equipment (Edinburgh Instruments Ltd., Edinburgh, UK), we obtained the PL and PLE spectra. A 405 nm laser was used to excite the transient PL, and an NIR photomultiplier (PMT928, Hamamatsu, Kyoto, Japan) was used to detect it. The effect of temperature on PL was investigated using a cryostat (OptistatDN2, Oxford Instruments, Abingdon, UK). Electron paramagnetic resonance (EPR) spectroscopy on a Bruker ESRA-300 (Bruker, Berlin-Adlershof, Germany) was used to analyze the Si dangling bond. Details of spectral analysis can be found in [14].

## 3. Results and Discussion

Figure 1 shows the process for the fabrication of Er-hyperdoped Si QDs. Data in prior publications have shown that 1% Er is a comparatively optimal concentration [15,16]. Nonthermal plasma, representative of far from thermal equilibrium, was used to produce Er-hyperdoped Si QDs with a 1% Er concentration (5 × 10^20^ cm^−3^) [17]. The QD surface was subsequently modified by hydrosilylation using 1-dodecene (Figure 1b) [18]. To put this in perspective, the Er solubility in crystalline Si is only ~10^18^ cm^−3^ [19], and therefore the Er concentration in this work was increased by two orders of magnitude. In the following, we discuss the 1% Er-hyperdoped Si QDs considered in this work.

**Figure 2:**
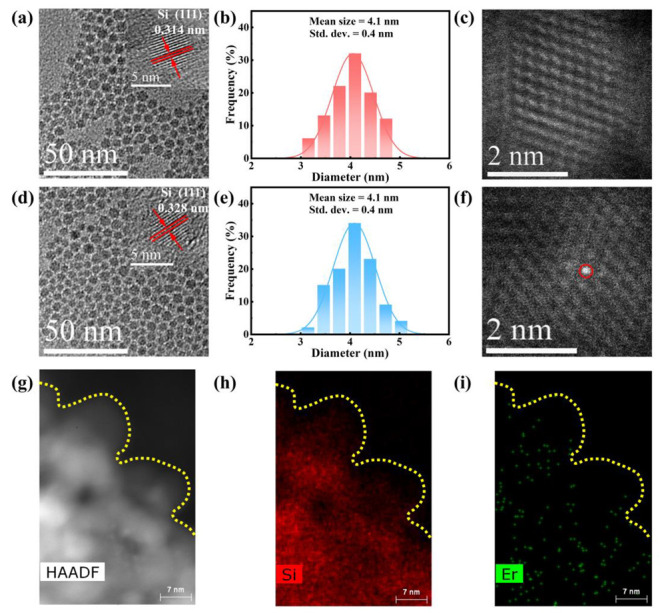
TEM images, size distribution, and HAADF-STEM images of (**a**–**c**) undoped and (**d**–**f**) Er-hyperdoped Si QDs. (**g**) HAADF-STEM image of Er-hyperdoped Si QDs and corresponding element maps for (**h**) Si and (**i**) Er.

The transmission electron microscopy (TEM) images of undoped and Er-hyperdoped Si QD are presented in Figure 2a,d. All of them show a sphere-like morphology, demonstrating that the morphological alteration induced by the hyperdoping of Er in Si QDs was negligible. The inset of Figure 2d shows a high-resolution TEM (HR-TEM) image of Er-hyperdoped Si QDs. The clear lattice fringe exhibits the excellent crystallinity in the Er-hyperdoped Si QDs. Moreover, good crystallinity was also observed in undoped Si QDs, as seen in the inset of Figure 2a. Figure 2a,d show that the Si (111) lattice spacing (d) increased from 0.314 nm for undoped Si QDs to 0.328 nm for Er-hyperdoped Si QDs. This may have resulted from the presence of sites of interstitial tetrahedral Er [20]. As shown in Figure 2b,e, the average diameters of both undoped and Er-hyperdoped Si QDs were 4.1 ± 0.4 nm. In addition, Figure 2f shows an HAADF-STEM image of an Er-hyperdoped Si QD, in which the brightest point is an Er atom. The Er atom was not present in undoped Si QDs (Figure 2c). The element maps indicate that that Er was closely associated to the Si element (Figure 2g–i), proving the Er atoms were incorporated into the Si QDs. Furthermore, the XRD patterns are presented in Figure S1. The pure diamond phases of Si QDs were detected in all samples, proving their high crystallinity (Figure S1). We thus draw the conclusion that hyperdoping did not alter the intrinsic diamond structure of Si QDs.

**Figure 3:**
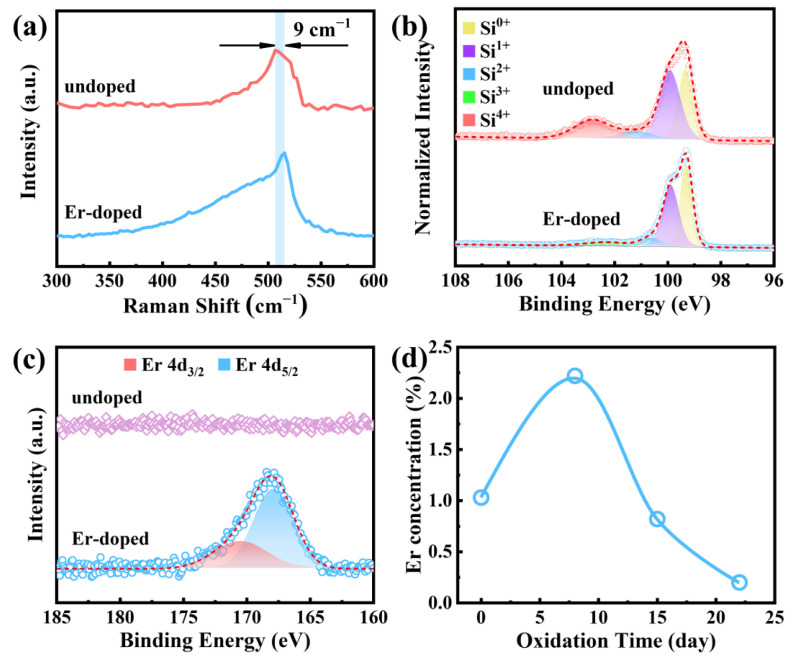
(**a**) Raman spectra, (**b**) Si 2p XPS spectra, and (**c**) Er 4d XPS spectra of Si QDs with pristine and Er hyperdoping. (**d**) Er concentration as a function of oxidation time.

In undoped Si QDs, Si-Si bonds were related to the Raman signal at ~506 cm^−1^ (Figure 3a). The phonon confinement effect is responsible for the redshift of the Raman signal for Si-Si bonds in bulk Si from its original position (~520 cm^−1^) [21]. The Si-Si bond Raman peak was found to have blueshifted from ~506 to 515 cm^−1^ after Er hyperdoping. This blueshift was brought on by the compressive strain caused by the presence of sites of tetrahedral interstitial Er [21]. It is reasonable to make an approximation of the compressive strain (*ε*) using [22]

(2) $$\varepsilon =\left(\frac{\Delta \nu }{691.2}\right)\times 100\%$$

where Δ*ν* denotes the shift of the Raman peak. We observed a 9 cm^−1^ shift in the Raman peak, which suggests *ε* = ~1.3%. On the other hand, Raman peak broadening of the Si-Si bonds suggests that these bonds were deformed when Er^3+^ was doped [22]. This provides more evidence that Er was present in the Si QDs. After 15 days of exposure in ambient air, Si 2p XPS spectra were obtained and are presented in Figure 3b. An in-depth analysis of the XPS data was conducted using the method given in [9]. The XPS data are consistent with the fact that Er hyperdoping generates a compressive strain in Si QDs [23], suggesting that Er greatly reduced oxidation in the Si QDs, as shown in Figure 3b. The surface SiO*_x_* thickness of Si QDs was reduced from 0.8 nm to 0.6 nm after Er hyperdoping, allowing for a corresponding SiO*_x_* stoichiometric shift from SiO_1.0_ to SiO_0.7_ (Table S1). XPS measurements of Er 4d are shown side by side in Figure 3c. Only the Er-hyperdoped Si QDs showed the characteristic binding energy peak corresponding to Er 4d. There have also been attempts to fit this Er 4d peak with mixed singlets. There is a 2 eV spin-orbit splitting in the Er 4d peak for Er-hyperdoped Si QDs [24], similar to that seen in Er_2_O_3_. As Er in Er_2_O_3_ is in the optically active valence of +3 [25], we may assume that the Er^3+^ was preserved throughout the hyperdoping process. The radial distribution of Er was evaluated after exposing Er-hyperdoped Si QDs to air at room temperature for varied durations of time. Figure 3d shows the Er concentration after the surface oxide had been etched away. Er concentrations clearly increased at the beginning of the oxidation process and decreased over time. Figure 3d shows that the maximum Er concentration of ~2.22% was attained after etching away the oxide produced over the period of 8 days. As a result, we can deduce that subsurface regions of the Si QDs may have contained the majority of the Er.

**Figure 4:**
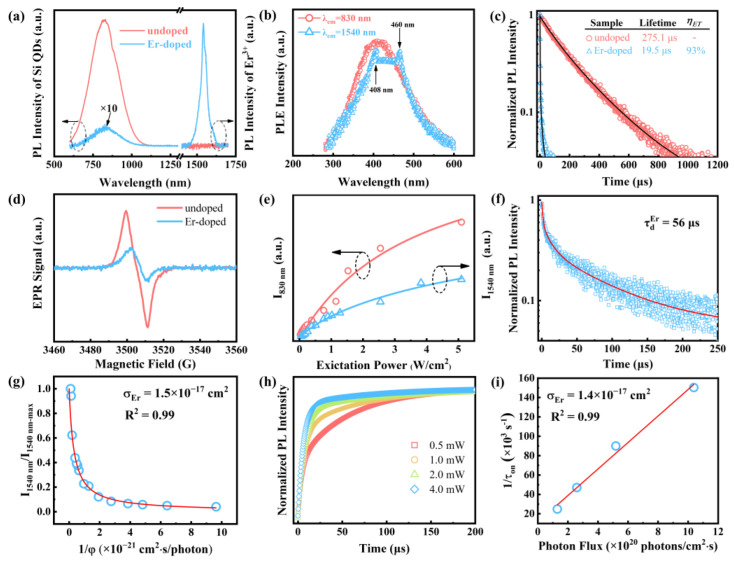
(**a**) NIR PL emission of Si QDs, pristine and with Er hyperdoping. (**b**) PLE spectra of Si QDs with Er hyperdoping. (**c**) The 830 nm PL decay curves of Si QDs, pristine and with Er hyperdoping. λ_ex_ = 405 nm. (**d**) First-derivative EPR spectra. (**e**) PL intensity detected at different excitation power, measured at 830 or 1540 nm. λ_ex_ = 405 nm. (**f**) PL decay monitored at 1540 nm (λ_ex_ = 405 nm) and the fitted curve. (**g**) The dependence of integrated PL intensity of the Er-related luminescence (1540 nm) on the reciprocal of photon flux. A line fitting the data is also shown. (**h**) Transient PL intensity at 1540 nm, normalized to the maximum value. (**i**) Photon-flux-dependent reciprocal of *τ_on_*.

As can be seen in Figure 4a, there was only one PL peak at 830 nm for undoped Si QDs. This peak was caused by the band gap transitions that occur in Si QDs [26,27]. Furthermore, Figure 2b,e show that undoped and Er-hyperdoped Si QDs had almost the same size distribution, resulting in invariable emission at 830 nm for undoped and Er-hyperdoped Si QDs. In addition, this result is commensurate with the quantum confinement effect [28,29]. On the other hand, following Er hyperdoping, a distinct PL peak appeared at 1540 nm. The 1540 nm emission is ascribable to the ^4^I_13/2_ → ^4^I_15/2_ transition of Er^3+^ [30]. In Figure 4a, a significant reduction in the intensity of the 830 nm emission after hyperdoping can be seen. Energy transfer to the Er^3+^ ions, previously seen in the literature, may account for this decrease [10]. In terms of the PL excitation (PLE) spectra, the peak for the 830 nm emission occurred at 405 nm, as shown in Figure 4b. This is because the exciton occupancy probability at 1.49 eV (λ = 830 nm) drops precipitously after exceeding 3.06 eV (i.e., λ < 405 nm) [31]. The PLE spectrum shows a broad peak for the 1540 nm emission, superimposed over the resonance peaks at 408 nm (^2^H_9/2_ → ^4^I_15/2_) and 460 nm (^4^F_5/2_ → ^4^I_15/2_). The synchronicity of the PLE spectrum for the 830 and 1540 nm emissions suggests that Er^3+^ was excited through an efficient energy transfer from Si QDs to Er^3+^, which is the critical attribute responsible for the emission of Er^3+^. The PL lifetime was shown to drop from 275.1 s to 19.5 s due to Er hyperdoping (Figure 4c and Note S1). Using the first-derivative EPR spectra in Figure 4d, we estimated that the area density of dangling bonds at the surface of undoped Si QDs was 1.3 × 10^12^ cm^−2^ (corresponding to 0.6 dangling bonds per QD) whereas that at the surface of Er-hyperdoped Si QDs was 6.9 × 10^11^ cm^−2^ (corresponding to 0.4 dangling bonds per QD) (details can be found in [14]). Hence, the decreased area density of dangling bonds excluded the contribution of surface dangling bonds to the decrease of the PL lifetime.

It was shown that the radiative recombination lifetime of Si QDs was not strongly influenced by compression because the compression hardly changed the HOMO–LUMO transition [32]. This means that the compressive strain hardly affected the PL lifetime. This suggest that the decrease of the PL lifetime is mainly induced by the transfer of energy from a Si QD to Er^3+^, assuming that no other factors play a role. Using Equation (1), we obtain an ultrahigh *η_ET_* of ~93%, which is substantially larger than the previously reported ~50% for Si QDs with neighboring Er^3+^ [10,11,33].

As seen in Figure 4e, the intensity of the PL at 830 nm (I_830 nm_) increased linearly with the increase of excitation power until the excitation power was so high that the PL tended to be saturated. For Si QDs, Auger recombination is responsible for this saturation [34]. Like I_830 nm_, the PL intensity at 1540 nm (I_1540 nm_) was proportional to the power of the 405 nm laser. Since this indicates that the I_1540 nm_ is constrained by the total amount of excitons transferred from Si QDs, this observation provides more evidence for the existence of excitonic energy transfer to Er^3+^ in Si QDs. We can calculate the effective excitation cross section of Er^3+^ (*σ_Er_*) using [35]

(3) $$\frac{I_{1540\;\mathrm{nm}}}{I_{1540\;\mathrm{nm}-max}}=\frac{1}{1+\frac{1}{\sigma_{Er}\tau_{d}^{Er}}.\frac{1}{\varphi }}$$

where *φ* represents the photon flux and $\tau_{d}^{Er}$ is the total lifetime of Er^3+^ in level ^4^I_13/2_. By fitting the data in Figure 4f,g, the value of *σ_Er_* was obtained as 1.5 × 10^−17^ cm^2^, an increase by four orders of magnitude compared with the value obtained for the direct excitation of Er^3+^ (8 × 10^−21^ cm^2^) [35]. The following equation describes the rise time (*τ_on_*) obtained for the 1540 nm light emission [36]

(4) $$\frac{1}{\tau_{on}}=\sigma_{Er}\varphi +\frac{1}{\tau_{d}^{Er}}$$

Hence, we can calculate *σ_Er_* by fitting Equation (4). We monitored the excitation-power-dependent rise for the 1540 nm light emission over time (Figure 4h). Figure 4i shows the fitting results, leading to a value of 1.4 × 10^−17^ cm^2^ for *σ_Er_*, which is quite similar to the result obtained using the data in Figure 4g. By assuming a strong coupling between a Si QD and Er^3+^, we were able to derive a consistent description on the ultrahigh *η_ET_* and *σ_Er_*. The coupling constant (*γ*) between a Si QD and Er^3+^ was calculated using the formula in the literature [37] with the assumption of *σ_Si QD_* = 10^−16^ cm^2^, *φ* = 10^20^ cm^−2^·S^−1^, $\tau_{d}^{Si\;QD}$ = 10^−5^ s, $\tau_{d}^{Er}$ = 5.6 × 10^−5^ s and *A_0_* < 10^17^ cm^−3^, where *σ_Si QD_* is the absorption cross section of Si QD, $\tau_{d}^{Si\;QD}$ is the Si QD PL lifetime, and A_0_ is the initial state of the Si QD. Finally, we obtained that the *γ* was comparable to or greater than the value of 1.8 × 10^−12^ cm^3^·s^−1^, an increase by three orders of magnitude compared with the *γ* in Er-doped silicon-rich silica (3 × 10^−15^ cm^3^·s^−1^) [36]. Therefore, the strong coupling between Si QDs and Er^3+^ allows Er^3+^ to be efficiently excited, resulting in the ultrahigh *η_ET_* and *σ_Er_*.

**Figure 5:**
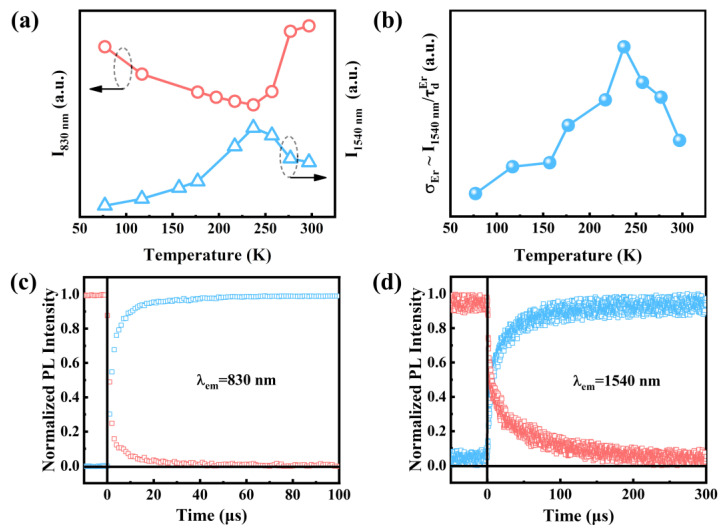
(**a**) Temperature dependence of the integrated PL intensity. (**b**) Temperature dependence of $\sigma_{Er}\sim I_{1540\;\mathrm{nm}}/\tau_{d}^{Er}$. PL rise time and decay time for (**c**) Si QDs (830 nm) and (**d**) Er^3+^ (1540 nm) at a pump power of 2 mW.

The dependence of the integrated PL intensity on temperature was studied to further verify the strong coupling. Figure 5a shows that I_830 nm_ decreased with the increase of temperature from 77 to 237 K while it increased with the increase of temperature from 237 K to room temperature. However, there was only a monotonic decrease in I_830 nm_ with the increase of temperature in undoped Si QDs (Figure S2). Thermal quenching accounts for the decrease of I_830 nm_ between 77 K and 237 K [38]. In contrast, I_830 nm_ increased as the temperature increased from 237 K to room temperature due to the phonon-mediated energy backtransfer from Er^3+^ [39]. Please note that the reverse trend was found for the I_1540 nm_. In the weak excitation regime, we have [40]

(5) $$I\;\sim \;\sigma_{Er}\varphi N_{Er}\frac{\tau_{d}^{Er}}{\tau_{rad}^{Er}}$$

where *N_Er_* is the excited Er^3+^ concentration and $\tau_{rad}^{Er}$ is the radiative lifetime of excited Er^3+^. Furthermore, assuming that *N_Er_* and $\tau_{rad}^{Er}$ are temperature-independent, *σ_Er_* can be obtained by Equation (5). Thus, *σ_Er_* is proportional to $I/\tau_{d}^{Er}$. As shown in Figure 5b, the temperature dependence of $\sigma_{Er}\sim I/\tau_{d}^{Er}$ has the same tendency as I_1540 nm_. Therefore, the temperature-dependent *σ_Er_* results in the trend of I_1540 nm_ with the increase of temperature. Moreover, the reverse temperature-dependent PL tendency between I_830 nm_ and I_1540 nm_ demonstrates the effective energy transfer of excitons between Si QDs and Er^3+^, further supporting the strong coupling mechanism.

Both the rise time (*τ_on_*) and decay time (*τ_d_*) of Si QDs (Figure 5c) and Er^3+^ (Figure 5d) were monitored in order to calculate the excitation rate. The excitation rate, denoted by *R* = (1*/τ_on_* − 1*/τ_d_*), was calculated, showing that *R_Si QD_* = 7962 s^−1^ for Si QDs and *R_Er_* = 7768 s^−1^ for Er^3+^. The observed *R_Er_* is close to *R_Si QD_*, which is also in agreement with the strong coupling mechanism [33].

## 4. Conclusions

The synthesis of Er-hyperdoped Si QDs was accomplished by employing nonthermal plasma. Both 830 and 1540 nm NIR light were emitted from Er-hyperdoped Si QDs under 405 nm excitation. PLE and PL decay measurements showed that energy transfer occurred between Si QDs and Er^3+^. For Er-hyperdoped Si QDs, an ultrahigh *η_ET_* and a high *σ_Er_* were obtained. Moreover, the quantitative study of the coupling constant demonstrates the strong coupling between Si QDs and Er^3+^. The strong coupling was also manifested by the temperature-dependent PL and excitation rate of Er-hyperdoped Si QDs. All these findings suggest that Er-hyperdoped Si QDs have great potential for the fabrication of high-performance NIR light-emitting devices.

## Data Availability

The data that support the findings of this study are available from the corresponding author upon reasonable request.

## Supplementary Materials

The following supporting information can be downloaded at: https://www.mdpi.com/article/10.3390/nano13020277/s1, Figure S1: XRD patterns of Si QDs, pristine and with Er hyperdoping; Figure S2: Temperature-dependent integrated emission intensities of undoped Si QDs; Table S1: Atomic fraction of various charge states of Si; Note S1: The fitting of PL decay curves and the determination of PL lifetime. Reference [9] is cited in the supplementary materials.
