# Supplementary material for: Highly Efficient Energy Transfer from Silicon to Erbium in Erbium-Hyperdoped Silicon Quantum Dots
Source: Nanomaterials (Basel). 2023 Jan 9;13(2):277. doi: 10.3390/nano13020277 (PMC9860861; doi:10.3390/nano13020277)
Supplement: Supplementary file 1 [file nanomaterials-13-00277-s001.zip › nanomaterials-2130271-supplementary.pdf]

Supplementary Materials

Highly Efficient Energy Transfer from Silicon to Erbium in Erbium-Hyperdoped Silicon Quantum Dots

Kun Wang [1], Qiang He [1], Deren Yang [1,2] and Xiaodong Pi [1,2,*]

[1] State Key Laboratory of Silicon and Advanced Semiconductor Materials & School of Materials Science and Engineering, Zhejiang University, Hangzhou 310027, China
[2] Institute of Advanced Semiconductors & Zhejiang Provincial Key Laboratory of Power Semiconductor Materials and Devices, Hangzhou Innovation Center, Zhejiang University, Hangzhou 311215, China
* Correspondence: xdpi@zju.edu.cn

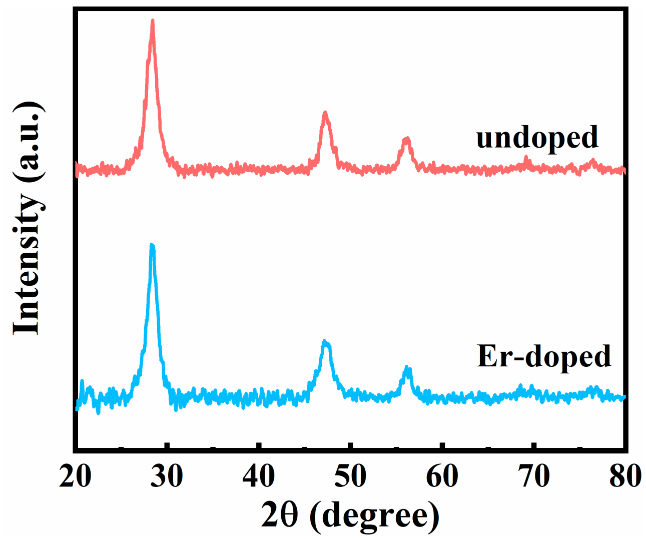

Figure S1. XRD patterns of Si QDs with pristine and Er hyperdoping.

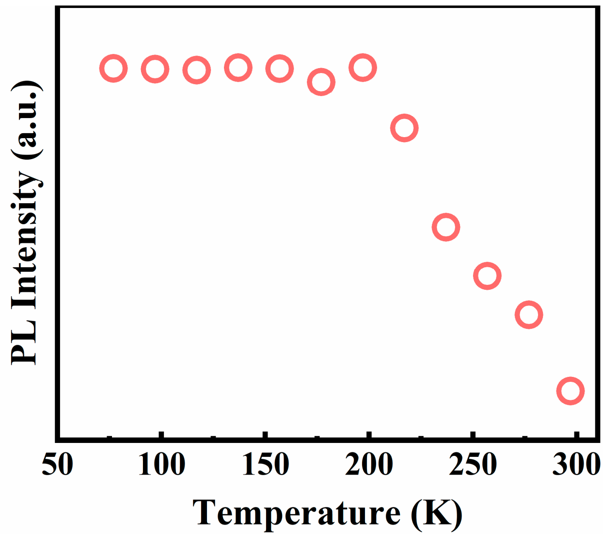

Figure S2. Temperature-dependent integrated emission intensity of undoped Si QDs.

Table S1. Atomic fraction of various charge states of Si

**Sample**	**$N_{Si^{0+}}$**	**$N_{Si^{1+}}$**	**$N_{Si^{2+}}$**	**$N_{Si^{3+}}$**	**$N_{Si^{4+}}$**	***SiO_x***	***d (nm)***
Undoped Si QDs	30.29	42.93	7.11	0	19.56	$SiO_{1.0}$	0.8
Er-hyperdoped Si QDs	41.98	43.21	7.38	7.43	0	$SiO_{0.7}$	0.6

Note S1. The fitting of PL decay curves and the determination of PL lifetime

Both of the PL decay curves can be well-fitted by a biexponential equation

$$I(t) = A_1 e^{-\frac{t}{\tau_1}} + A_2 e^{-\frac{t}{\tau_2}}, \quad \text{(S1)}$$

where $I(t)$ is the PL intensity at time t, A_1 and A_2 are fitting constants, and τ_1 and τ_2 are the fast and slow decay time, respectively. The effective average lifetime τ can be estimated by the following equation [9]

$$\tau = \frac{A_1 {\tau_1}^2 + A_2 {\tau_2}^2}{A_1 \tau_1 + A_2 \tau_2}, \quad \text{(S2)}$$

References

9. Wang, K.; He, Q.; Yang, D.; Pi, X. Erbium-Hyperdoped Silicon Quantum Dots: A Platform of Ratiometric Near-Infrared Fluorescence. *Adv. Opt. Mater.* **2022**, *10*, 2201831, doi:10.1002/adom.202201831.
